# A Foreword to Fellow-Workers

**Published:** 1914-10-10

**Authors:** 


### FOREWORD TO FELLOW-WORKERS.

The outbreak of war, with the part which the voluntary hospitals have played so unanimously and promptly, has brought home to our readers, not merely the work accomplished, but what active co-operation on their part and ours can achieve through the columns of The Hospital. At an exceptionally laborious time our special correspondents in the institutions have presented us week by week with vivid personal accounts of the steps of preparation and the arrival of the wounded, which have made our columns a file to be referred to after the war is over by all interested in this aspect of the great crisis through which the nation is passing. That co-operation could hardly have been achieved had not the whole field of voluntary hospital enterprise been accompanied of late years by a marked thirst for knowledge, which, as we show on another page in reference to the growth of hospital literature, has produced writers and readers on hospital subjects in increasing quantities. The training of hospital workers and of all interested in the support and management of these institutions has grown so many-sided of recent years that in each department every officer, whether lay committee-man or trained administrator, is feeling the need for the special discussion of his own problems.

The reader who casts a glance over the whole range of matters covered by this Special Number will realise how alive each department of a modern hospital is, and how great is the need for mutual exchange of information between similar departments in different institutions. The one danger which a large hospital in a modern city runs is that of a pre-occupation with its immediate affairs so great as to exclude a knowledge of what is going on in other centres. The competitions which we have recently started with a view to bringing out the best technical features in equipment possessed by different institutions illustrate convincingly the advantage of breaking through this barrier of self-sufficiency. It is important to recognise this, when matters of policy and principle, especially at the present time, are constantly arising; and at the beginning of our new volume we would urge every reader to co-operate with us by sending prompt information of every development or difficulty as it occurs. With the inspiration lately afforded, our readers and ourselves can contemplate the beginning of the new volume with high hopes for its interest and value.

